# Fibroblast-Derived PLA2G12A Amplifies Mast Cell Activation Through Extracellular Phospholipid Remodeling

**DOI:** 10.3390/cells15201848

**Published:** 2026-10-12

**Authors:** Yoshitaka Taketomi, Yoshimi Miki, Takayoshi Higashi, Yuki Nagasaki, Kuniyuki Kano, Makoto Arita, Junken Aoki, Makoto Murakami

**Affiliations:** 1 Laboratory of Microenvironmental and Metabolic Health Science, Center for Disease Biology and Integrative Medicine (CDBIM), Graduate School of Medicine, The University of Tokyo, 7-3-1 Hongo, Bunkyo-ku, Tokyo 113-8655, Japan; 2 Lipid Metabolism Project, Tokyo Metropolitan Institute of Medical Science, 2-1-6 Kamikitazawa, Setagaya-ku, Tokyo 156-8506, Japan; 3 Department of Health Chemistry, Graduate School of Pharmaceutical Sciences, The University of Tokyo, 7-3-1 Hongo, Bunkyo-ku, Tokyo 113-0033, Japan; 4 Laboratory for Metabolomics, RIKEN Center for Integrative Medical Sciences, 1-7-22 Suehiro-cho, Tsurumi-ku, Yokohama City 230-0045, Kanagawa, Japan; 5 Division of Physiological Chemistry and Metabolism, Graduate School of Pharmaceutical Sciences, Keio University, 1-5-30 Shiba-koen, Minato-ku, Tokyo 105-8512, Japan; 6 Human Biology-Microbiome-Quantum Research Center (WPI-Bio2Q), Keio University, 35 Shinanomachi, Shinjuku-ku, Tokyo 160-8582, Japan

**Keywords:** anaphylaxis, mast cell, fibroblast, phospholipase A_2_, lysophospholipid, extracellular vesicle

## Abstract

**Highlights:**

**What are the main findings?**

- Fibroblast-derived PLA2G12A promotes IgE/antigen-dependent mast cell activation and anaphylactic responses in vivo.
- PLA2G12A hydrolyzes phospholipids in the extracellular vesicle-enriched fraction to generate lysophospholipids that potentiate mast cell activation.

**What are the implications of the main findings?**

- Secreted phospholipase A_2_-driven extracellular phospholipid remodeling represents a mechanism for stromal regulation of mast cell responsiveness.
- Extracellular vesicle-enriched fractions contain potential phospholipid substrates for PLA2G12A.

**Abstract:**

Extracellular vesicles (EVs) mediate intercellular communication, yet whether extracellular phospholipid metabolism on EV membranes regulates immune activation remains incompletely understood. Through comparative phenotypic analyses of multiple secreted phospholipase A_2_ (sPLA_2_)-deficient mouse strains, we identified PLA2G12A as a stromal regulator of extracellular lipid metabolism that promotes IgE/antigen-dependent mast cell activation. *Pla2g12a* deficiency attenuated passive cutaneous and systemic anaphylaxis and selectively impaired IgE-dependent, but not IgE-independent, mast cell responses. Single-cell RNA-sequencing of mouse and human skin revealed that PLA2G12A is expressed in broad subsets of stromal fibroblasts, including papillary fibroblasts adjacent to mast cells, implicating a specialized stromal niche in mast cell regulation. Consistently, mast cell-intrinsic PLA2G12A was dispensable, whereas fibroblast-targeted deletion of *Pla2g12a* phenocopied global *Pla2g12a* deficiency. Lipidomic analyses during IgE-mediated anaphylaxis revealed a significant reduction in lysophospholipids, accompanied by minimal changes in oxylipins, in *Pla2g12a*-deficient skin. Mechanistically, fibroblast-derived PLA2G12A hydrolyzed phospholipids in EV-enriched fractions to generate multiple lysophospholipids, and treatment with either the PLA2G12A-treated EV-enriched fraction or exogenous lysophospholipids restored impaired IgE-dependent anaphylactic responses in *Pla2g12a*-deficient mice. Collectively, these findings establish sPLA_2_-driven extracellular phospholipid remodeling as a mechanism by which stromal niches regulate allergic inflammation.

## 1. Introduction

Allergic diseases, including asthma and atopic dermatitis, have become increasingly prevalent in recent decades. Environmental and lifestyle factors that disrupt epithelial barrier integrity and immune homeostasis promote IgE production and the activation of mast cells (MCs) via crosslinking of the high-affinity IgE receptor FcεRI by IgE and antigen (Ag) [1]. As tissue-resident immune cells, MCs orchestrate immediate hypersensitivity reactions and contribute to type 2 inflammatory diseases [2]. In addition to IgE/Ag-dependent FcεRI signaling, MC activation is profoundly influenced by the tissue microenvironment, which comprises stromal cells, extracellular matrix components, cytokines and lipid mediators [3,4]. Increasing evidence indicates that the stromal niche actively regulates MC activation and responsiveness in peripheral tissues.

Extracellular vesicles (EVs) have emerged as important mediators of intercellular communication in tissue microenvironments. EVs transport proteins, nucleic acids, and lipids that influence the behavior of recipient cells [5]. Since MCs rapidly release EVs upon IgE/Ag stimulation, EV-mediated signaling has been thought to modulate allergic responses [6,7]. Beyond their role as carriers of protein and nucleic acid cargos, EV membranes are enriched in phospholipids that can serve as substrates for extracellular lipid-metabolizing enzymes [8,9]. Recent studies have revealed that EV-associated phospholipids are not merely structural components of vesicle membranes but also serve as precursors for active mediators of intercellular communication. Extracellular hydrolysis of EV phospholipids generates bioactive lipid mediators, including lysophospholipids and fatty acid-derived metabolites (oxylipins), which ultimately regulate diverse biological processes such as immune and inflammatory responses, tumor progression, MC maturation, and pathogenic T cell differentiation [10,11,12,13,14,15].

Lipid mediators are critically involved in the regulation of MC biology and allergic inflammation. Upon activation, MCs produce arachidonic acid (AA)-derived oxylipins called eicosanoids, including prostaglandin D_2_ (PGD_2_) and leukotriene C_4_ (LTC_4_), which amplify type 2 immune responses and allergic inflammation [16,17,18]. Phospholipase A_2_ (PLA_2_) enzymes hydrolyze membrane phospholipids at the *sn*-2 position to release free fatty acids and lysophospholipids, thereby initiating diverse lipid signaling pathways [19,20]. The production of these eicosanoids in activated MCs is initiated by cytosolic phospholipase A_2_α (cPLA_2_α; PLA2G4A), which selectively hydrolyzes AA-containing phospholipids to supply the substrate for eicosanoid biosynthesis [20,21,22,23]. In addition, MCs express platelet-activating factor acetylhydrolase II (PAFAH2; PLA2G7B), an intracellular PLA_2_ preferentially hydrolyzing oxidized phospholipids to liberate ω3 polyunsaturated fatty acid (PUFA)-derived epoxy lipids, which optimize FcεRI-dependent MC activation and immediate allergic responses [24]. Beyond serving as a source of lipid mediators, MCs are themselves regulated by diverse extracellular lipid signals, including PUFAs, lysophospholipids, sphingolipids, and their metabolites, which control multiple aspects of MC biology, including homing, maturation and activation [12,25,26,27,28,29].

Among the secreted phospholipase A_2_ (sPLA_2_) family, which comprises 11 isoforms with distinct tissue distributions and substrate preferences, we have identified group III sPLA_2_ (PLA2G3), the sole mammalian homolog of bee venom PLA_2_, as a critical regulator of MC maturation [12,18,29,30,31]. MC-secreted PLA2G3 acts on EV membrane phospholipids and, through functional coupling with neighboring fibroblasts, promotes fibroblast-dependent lysophosphatidic acid (LPA) signaling, thereby driving MC maturation and associated anaphylactic responses [12,29,31]. These findings highlight that sPLA_2_-driven extracellular phospholipid metabolism is an important regulator of allergic inflammation. Notably, several sPLA_2_ isoforms, when overexpressed or added exogenously at high concentrations, have been reported to promote MC activation in vitro [29,32,33,34]. However, beyond the regulation of MC maturation by the sPLA_2_-III–EV axis as mentioned above [12,29,31], it remains unclear whether extracellular lipid metabolism mediated by endogenous sPLA_2_s would contribute to MC activation in vivo. Furthermore, although several lysophospholipids, such as lysophosphatidylserine (LysoPS), have been shown to potentiate FcεRI-dependent MC degranulation [25,35], it remains unknown whether sPLA_2_-dependent lysophospholipid production constitutes a physiological pathway regulating MC activation, and if so, which sPLA_2_ isoform and lysophospholipid species are responsible for this process. In particular, whether EV membrane phospholipids could serve as functional substrates for an endogenous sPLA_2_ to regulate MC activation during allergic responses has not yet been explored.

Here, we identify PLA2G12A, an atypical sPLA_2_ isoform [36,37], as a stromal regulator of IgE-dependent MC activation, but not MC maturation. Through comprehensive phenotypic analyses of multiple sPLA_2_-deficient mouse strains, followed by lipidomics, EV reconstitution, and fibroblast-targeted gene deletion, we provide evidence that fibroblast-derived PLA2G12A facilitates IgE-dependent MC activation and immediate hypersensitivity responses through extracellular phospholipid remodeling.

## 2. Materials and Methods

### 2.1. Mice

Various PLA_2_-deficient mouse strains on a C57BL/6 background have been described previously [14,38,39,40,41,42,43,44]. MC-deficient *Kit^W-sh/W-sh^* mice (JAX stock #005051) were purchased from the Jackson Laboratory (Bar Harbor, ME, USA) [45]. *Pla2g12a* floxed mice [46] were crossed with *Col1a2*-Cre transgenic mice (Resource No. nbio228; Laboratory Animal Resource Bank, National Institutes of Biomedical Innovation, Health and Nutrition, Ibaraki, Osaka, Japan) [47] to generate fibroblast-targeted *Pla2g12a* conditional knockout mice (*Pla2g12a*^fl/fl^ *Col1a2*-Cre). Male mice aged 8–12 weeks were used in all experiments, except for an additional experiment performed using female mice of the same age range. Mice were housed under specific pathogen-free conditions on a 12-h light-dark cycle with free access to food and water. Animal experiments were performed at the Tokyo Metropolitan Institute of Medical Science and the University of Tokyo. All animal experiments were approved by the institutional animal care and use committees and conducted in accordance with the Japanese Guide for the Care and Use of Laboratory Animals.

### 2.2. Anaphylaxis

IgE-dependent passive cutaneous (PCA) or systemic (PSA) anaphylaxis induced by Ag and IgE-independent cutaneous anaphylaxis induced by compound 48/80 (C48/80) were performed as described previously [12,29,31]. For PCA, mice were sensitized by intradermal injection of anti-DNP IgE (SPE-7, Sigma-Aldrich, St. Louis, MO, USA; 30 ng) into the ear pinna. Twenty-four hours later, mice were challenged intravenously with dinitrophenylated human serum albumin (DNP-HSA, Sigma-Aldrich; 60 µg) and Evans blue (FUJIFILM Wako, Osaka, Japan; 1 mg per mouse). For IgE-independent cutaneous anaphylaxis, C48/80 (Sigma-Aldrich; 250 ng) was injected intradermally into the ear, followed immediately by intravenous Evans blue administration. Ear swelling was assessed by measuring ear thickness before and 30 min after challenge using a micrometer (Mitutoyo, Kawasaki, Japan). Vascular permeability was quantified by spectrophotometric measurement at 620 nm of Evans blue extracted from IgE-sensitized or phosphate-buffered saline (PBS)-treated ears collected 30 min after Ag challenge. For PSA, mice were sensitized intravenously with anti-DNP IgE (3 µg per mouse) and challenged the following day with DNP-HSA (500 µg per mouse). Rectal temperature was measured over time using a digital thermometer with a rectal probe (Physitemp Instruments, Clifton, NJ, USA).

### 2.3. Measurement of Histamine

Histamine levels in sera and tissues were measured by LC-MS/MS with minor modifications of a previously described method [12]. Tissues were homogenized using a Precellys Homogenizer (Bertin Technologies, Montigny-le-Bretonneux, France) in Hank’s balanced salt solution (FUJIFILM Wako) containing 0.5% (*v*/*v*) Triton X-100, followed by perchloric acid extraction. Serum samples were mixed with methanol and centrifuged to remove precipitated proteins. Deuterium-labeled histamine·2HCl (α,α,β,β-D_4_) (Cambridge Isotope Laboratories, Tewksbury, MA, USA) was used as an internal standard for quantification.

### 2.4. Measurement of MC Protease Activities

MC protease activity in tissue extracts was measured as described previously [48]. Tissue extracts prepared for histamine measurement (see above) were used to evaluate protease activity. Chymotrypsin-like, trypsin-like, and carboxypeptidase A (CPA) activities were measured using the chromogenic substrates S-2586 (MeO-Suc-Arg-Pro-Tyr-pNA; DiaPharma, West Chester, OH, USA), S-2288 (H-D-Ile-Pro-Arg-pNA-2HCl; DiaPharma), and M-2245 (N-(4-methoxyphenylazoformyl)-Phe-OH potassium salt; Bachem, Bubendorf, Switzerland), respectively. Absorbance at 405 nm was measured before and after incubation at 37 °C, and protease activities were calculated from the change in absorbance per minute. Each activity was normalized to total protein quantity.

### 2.5. Histology

Ear pinnae were collected and fixed in 10% (*v*/*v*) Neutral Buffered Formalin (FUJIFILM Wako). Ear tissues were embedded in paraffin using an automated tissue processor (Tissue-Tek VIP 5 Jr; Sakura Finetek Japan, Tokyo, Japan) and sectioned at 4 μm using a sliding microtome (REM-710; YAMATO KOHKI, Niiza, Saitama, Japan). The sections were stained with 0.05% (*w*/*v*) toluidine blue (pH 0.5) (FUJIFILM Wako), and images were acquired using an All-in-One Fluorescence Microscope (BZ-X710; KEYENCE, Osaka, Japan). Toluidine blue-positive metachromatic cells were quantified as MCs.

### 2.6. Preparation, Maturation, and Activation of Bone Marrow-Derived Mast Cells

Bone marrow-derived mast cells (BMMCs) were generated and activated with minor modifications of a previously described method [12,29,49]. Briefly, bone marrow cells isolated from mouse femurs were cultured in Dulbecco’s modified Eagle’s medium (DMEM) containing 10% (*v*/*v*) fetal bovine serum (FBS; Biosera, Cholet, France) and baculovirus-derived recombinant mouse IL-3 (10 ng/mL) [50]. For maturation into connective tissue-type mast cell (CTMC)-like cells, BMMCs were cocultured with Swiss 3T3 fibroblasts (JCRB9019, Japanese Collection of Research Bioresources, Ibaraki, Osaka, Japan) in the presence of stem cell factor (SCF, 100 ng/mL; PeproTech, Cranbury, NJ, USA) for 4 days, with half of the culture medium replaced every 2 days. MCs were then separated from fibroblasts by replating or magnetic sorting using anti-CD117 (c-Kit) microbeads (Miltenyi Biotec, Bergisch Gladbach, Germany). MC maturation was assessed by histamine content, expression of mast cell marker genes, and cell-surface expression of FcεRIα and c-Kit. For activation assays, BMMCs (10^6^ cells) were sensitized with anti-DNP IgE (1 µg/mL) for 2 h, washed, and stimulated with DNP-BSA (100 ng/mL; Sigma-Aldrich) at 37 °C. Alternatively, the cells were stimulated with C48/80 (10 µg/mL).

### 2.7. β-Hexosaminidase Release Assay

IgE-sensitized BMMCs were stimulated with DNP-BSA at 37 °C for 10 min. Culture supernatants and cell pellets were collected separately, and β-hexosaminidase activity was measured using *p*-nitrophenyl *N*-acetyl-β-D-glucosaminide (FUJIFILM Wako) as a substrate. β-Hexosaminidase release was calculated as S/(S + P) × 100, where S and P represent β-hexosaminidase activities in the culture supernatant and the cell pellet fractions, respectively.

### 2.8. Measurement of Lipid Mediators and Cytokines

IgE-sensitized BMMCs were stimulated with DNP-BSA at 37 °C. For lipid mediator analysis, culture supernatants were collected after 10 min, and PGD_2_ and LTC_4_ levels were measured using the PGD_2_-MOX ELISA Kit and LTC_4_ ELISA Kit (both from Cayman Chemical, Ann Arbor, MI, USA), respectively. For cytokine analysis, culture supernatants were collected after 4 h, and TNF-α levels were quantified using a TNF-α ELISA Kit (R&D Systems, Minneapolis, MN, USA) according to the manufacturer’s instructions.

### 2.9. Reverse Transcription-Quantitative PCR (RT-qPCR)

For stimulation experiments, IgE-sensitized BMMCs were stimulated with DNP-BSA at 37 °C for 30 min before RNA isolation. For coculture experiments, BMMCs were cocultured with Swiss 3T3 fibroblasts in the presence of SCF for 4 days as described above. Total RNA was extracted from tissues and cultured cells using TRIzol Reagent (Invitrogen, Carlsbad, CA, USA). cDNA was synthesized using the High-Capacity cDNA Reverse Transcription Kit (Applied Biosystems, Foster City, CA, USA). Quantitative PCR (qPCR) was performed using the TaqMan Gene Expression Master Mix and predesigned TaqMan probe-primer assays on the StepOnePlus Real-Time PCR System (Applied Biosystems). Detailed information on the primer-probe sets used is provided in Table S1. Gene expression levels were normalized to *Gapdh* (Mouse GAPDH Endogenous Control; Applied Biosystems) using the 2^−ΔΔCt^ method and are presented relative to the control group. Where indicated, mast cell-associated genes were further normalized to *Kit* expression to account for differences in MC abundance.

### 2.10. MC Reconstitution

BMMCs (10^6^ cells) were injected intradermally into the ears of 6-week-old MC-deficient *Kit^W-sh/W-sh^* mice for MC reconstitution [12,29,31,45]. Mice were analyzed 6 weeks after cell transfer.

### 2.11. Preparation of Single-Cell Suspensions from Ear Skin

Ear skin was minced and treated with Accutase (Innovative Cell Technologies, San Diego, CA, USA), followed by digestion with Liberase TM (250 µg/mL; Roche, Mannheim, Germany) and DNase I (2 Kunitz units/mL; Sigma-Aldrich) in RPMI-1640 containing 10 mM HEPES and 5% FBS. Liberase/DNase digestion was performed twice. Cells were enriched by 25% (*v*/*v*) Percoll density-gradient centrifugation and passed through a 40-µm cell strainer to obtain single-cell suspensions. Single-cell suspensions were used for flow cytometry, RT-qPCR, or CD140a^+^ cell enrichment using the mouse CD140a (PDGFRα) MicroBead Kit (Miltenyi Biotec, Bergisch Gladbach, Germany).

### 2.12. Flow Cytometry

Tissue-derived single-cell suspensions and cultured BMMCs were resuspended in a FACS buffer consisting of Ca^2+^/Mg^2+^-free PBS containing 1% (*w*/*v*) BSA and 2 mM EDTA. Cells were incubated with TruStain FcX (anti-mouse CD16/32; BioLegend, San Diego, CA, USA) on ice for 10 min prior to antibody staining. The cells were then stained with fluorochrome-conjugated monoclonal antibodies against mouse CD117 (c-Kit) FITC (2B8; BD Pharmingen, San Diego, CA, USA), FcεRIα PE (MAR-1; eBioscience, San Diego, CA, USA), and CD45 Brilliant Violet 421 (30-F11; BioLegend). Corresponding isotype controls were used where indicated. Flow cytometry was performed using a FACSMelody Cell Sorter (BD Biosciences, San Jose, CA, USA), and data were analyzed using FlowJo v10.10.1 software (BD Biosciences). MCs were defined as CD45^+^CD117^+^FcεRIα^+^ cells in skin-derived single-cell suspensions and as CD117^+^FcεRIα^+^ cells in cultured BMMCs.

### 2.13. Single-Cell RNA-Sequencing (scRNA-Seq) Analysis

The mouse skin scRNA-seq dataset generated in our previous study [12] and deposited in Gene Expression Omnibus (GEO) under accession number GSE262558 was reanalyzed to assess *Pla2g12a* expression across skin cell populations. Fibroblast subclustering and visualization were performed using Seurat (v4.2.1). A published human skin scRNA-seq dataset (GSE241132) [51,52] was reanalyzed to examine *PLA2G12A* expression in fibroblast subsets. Fibroblasts were isolated, subclustered, and annotated on the basis of canonical marker-gene expression using Scanpy (v1.11.3).

### 2.14. Preparation of the EV-Enriched Fraction

EV-enriched fractions were prepared from fibroblast–MC coculture supernatants as described previously [12,53]. Because fibroblast–BMMC coculture in the presence of SCF recapitulates the niche environment that supports CTMC maturation and activation [4,12,54], EV-enriched fractions were prepared directly from coculture supernatants without additional IgE/Ag stimulation. Briefly, conditioned media were collected after 2 days of culture, filtered through 0.22-μm PVDF filters (Merck Millipore, Burlington, MA, USA), and concentrated using Vivaspin 20 Centrifugal Concentrators (Sartorius, Göttingen, Germany). Concentrated samples were mixed with ExoQuick-TC reagent (System Biosciences, Palo Alto, CA, USA) and incubated overnight at 4 °C. EV-enriched fractions were recovered by centrifugation at 6000× *g* for 30 min, washed once with Ca^2+^/Mg^2+^-free PBS, and collected by an additional centrifugation step at 6000× *g* for 15 min. Pellets were resuspended in the indicated buffer and subjected to subsequent analyses. As this isolation procedure relies on polymer-based precipitation rather than classical density-gradient or size-exclusion purification, the resulting material is referred to as the EV-enriched fraction throughout this study.

### 2.15. PLA_2_ Assay Using the EV-Enriched Fraction as a Substrate

For PLA_2_ reactions, the EV-enriched fraction corresponding to 2 × 10^6^ BMMC equivalents was incubated with recombinant human PLA2G12A protein, generously provided by Dr. Gérard Lambeau (Centre National de la Recherche Scientifique (CNRS), France), in reaction buffer containing 100 mM Tris-HCl (pH 7.5) and 5 mM CaCl_2_ in a total volume of 150 µL. After incubation at 37 °C for 30 min, reactions were terminated by the addition of methanol containing internal standards. Lipids were extracted by the Bligh and Dyer method [55] and analyzed by liquid chromatography–tandem mass spectrometry (LC-MS/MS) as previously described [12] using a 4000 QTRAP triple quadrupole linear ion trap mass spectrometer (SCIEX, Marlborough, MA, USA) coupled to a Nexera UPLC system (Shimadzu, Kyoto, Japan). Phospholipids, lysophospholipids, and free fatty acids were quantified by multiple reaction monitoring.

### 2.16. In Vivo Rescue Experiments with the PLA2G12A-Treated EV-Enriched Fraction and Lysophospholipids

For in vivo rescue experiments, the EV-enriched fraction was incubated with recombinant mouse PLA2G12A (300 ng/mL), and 30 µL of the preparation containing 8 or 18 µg of the EV-enriched fraction was administered intradermally into the ear immediately before Ag challenge. Since PLA2G12A was not removed or inactivated before administration and was therefore carried over, equivalent amounts of PLA2G12A alone and the EV-enriched fraction without PLA2G12A pretreatment were injected into the ear as controls. For rescue experiments using pure lysophospholipids, each of lysophosphatidylcholine (LPC), lysophosphatidylethanolamine (LPE), LysoPS, or LPA, all having an 18:1 acyl chain at the *sn*-1 position (Avanti Polar Lipids, Alabaster, AL, USA), was administered intradermally into the ear at doses of 0, 15, 150, or 300 pmol per ear immediately before Ag challenge.

### 2.17. Lipidomic Analysis

For in vivo lipidomic analysis, ear skin samples were collected from *Pla2g12a*^+/+^ and *Pla2g12a*^−/−^ mice before and 2 min after Ag challenge in the PCA model. Individual molecular species of LPC, LPE, LysoPS, LPA, LPG, and LPI were quantified by LC–MS/MS, as described previously [56]. Free fatty acid and oxylipin analyses were performed by the Laboratory for Metabolomics, RIKEN Center for Integrative Medical Sciences, using previously published methods [57,58].

### 2.18. Statistical Analysis

Statistical analyses were performed using GraphPad Prism software (v11.1.0; GraphPad Software, Boston, MA, USA). Data are presented as mean ± s.e.m. or as indicated in the figure legends. Animals were allocated to experimental groups without randomization, and investigators were not blinded during outcome assessment unless otherwise indicated. Comparisons between two groups were performed using an unpaired two-tailed Student’s *t*-test or Mann–Whitney U test, as appropriate. Comparisons among multiple groups were performed using ordinary one-way or two-way ANOVA followed by Šídák’s or Tukey’s multiple-comparisons test, or Brown–Forsythe and Welch ANOVA followed by Dunnett’s T3 multiple-comparisons test, as indicated in the figure legends. For lipidomic analyses involving multiple lipid species, *p* values were adjusted for multiple testing using the Benjamini–Hochberg false discovery rate (FDR) procedure, as indicated in the figure legends. A *p* value < 0.05 was considered statistically significant.

## 3. Results

### 3.1. PLA2G12A Promotes IgE/Ag-Dependent MC Activation In Vivo

**Figure 1:**
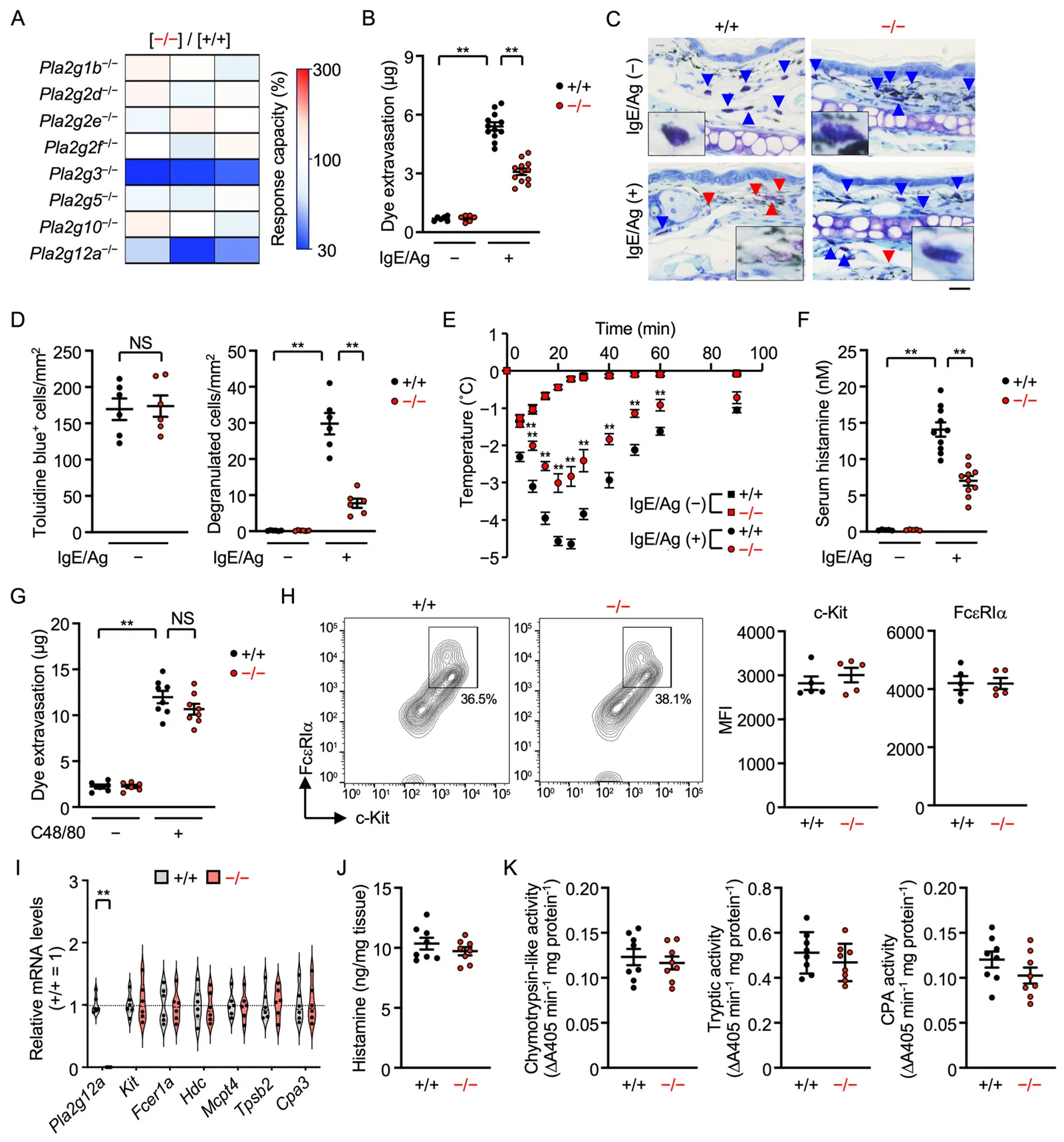
PLA2G12A deficiency attenuates IgE/Ag-dependent MC activation and anaphylactic responses in vivo. (**A**) Relative IgE/Ag-induced PCA responses in mouse strains deficient in individual sPLA_2_ isoforms. Vascular permeability was assessed by Evans blue extravasation. Colors indicate the responses of individual knockout mice (*n* = 3 per genotype) relative to those of littermate controls (+/+), which were set to 100%. (**B**) Evans blue extravasation under IgE/Ag (–) and IgE/Ag (+) PCA conditions in *Pla2g12a*^+/+^ and *Pla2g12a*^−/−^ mice. (**C**) Representative toluidine blue-stained ear sections from *Pla2g12a*^+/+^ and *Pla2g12a*^−/−^ mice under IgE/Ag (–) and IgE/Ag (+) conditions. Blue and red arrowheads indicate intact and degranulated MCs, respectively. Insets show higher-magnification views. Scale bar, 25 µm. (**D**) Quantification of total (left) and degranulated (right) MCs in ear sections shown in (**C**). (**E**,**F**) IgE/Ag-induced PSA in *Pla2g12a*^+/+^ and *Pla2g12a*^−/−^ mice. Changes in rectal temperature following Ag challenge (**E**) and serum histamine levels (**F**) are shown. *n* = 5 per genotype for IgE/Ag (–) and *n* = 9 per genotype for IgE/Ag (+) (**E**). (**G**) Evans blue extravasation in C48/80-induced cutaneous anaphylaxis in *Pla2g12a*^+/+^ and *Pla2g12a*^−/−^ mice. (**H**) Flow cytometric analysis of dermal MCs from ear skin of *Pla2g12a*^+/+^ and *Pla2g12a*^−/−^ mice. Representative contour plots (left) and quantification of surface c-Kit and FcεRIα expression (right) are shown. (**I**) Relative expression of mast cell marker genes in ear tissues from *Pla2g12a*^+/+^ and *Pla2g12a*^−/−^ mice. Expression levels in *Pla2g12a*^+/+^ mice were set to 1. (**J**,**K**) Histamine content (**J**) and chymotrypsin-like, trypsin-like, and CPA activities (**K**) in ear tissue extracts from *Pla2g12a*^+/+^ and *Pla2g12a*^−/−^ mice. Data are presented as mean ± s.e.m. Representative images from two independent experiments are shown in (**C**). Data are pooled from one (**H**–**K**), two (**D**) or three (**A**,**B**,**E**–**G**) independent experiments. Statistical analysis was performed using two-tailed Mann–Whitney U test (**A**,**H**–**K**), Brown–Forsythe and Welch ANOVA followed by Dunnett’s T3 multiple comparisons test (**B**,**D**,**F**,**G**), or two-way ANOVA followed by Tukey’s multiple comparisons test (**E**). **, *p* < 0.01; NS, not significant.

To assess whether sPLA_2_ isoforms would be involved in IgE/Ag-dependent MC activation in vivo, we performed PCA-based phenotypic screening using multiple sPLA_2_-deficient mouse strains, with Evans blue extravasation as a readout. In addition to the expected reduction in dye extravasation in *Pla2g3*^−/−^ mice, consistent with the established role of PLA2G3 in MC maturation [12,29,31], *Pla2g12a*^−/−^ mice, but not mice lacking other sPLA_2_s (*Pla2g1b*^−/−^, *Pla2g2d*^−/−^, *Pla2g2e*^−/−^, *Pla2g2f*^−/−^, *Pla2g5*^−/−^ and *Pla2g10*^−/−^), also exhibited a markedly attenuated PCA response (Figure 1A). IgE/Ag-induced Evans blue extravasation was significantly reduced in male *Pla2g12a*^−/−^ mice compared with littermate *Pla2g12a*^+/+^ controls (Figure 1B), as shown in a representative gross image of the ears 30 min after Ag challenge (Figure S1A). Female *Pla2g12a*^−/−^ mice also exhibited an attenuated PCA response compared with *Pla2g12a*^+/+^ mice (Figure S1B). Histological analysis of toluidine blue-stained ear sections revealed that, despite comparable dermal MC abundance between *Pla2g12a*^+/+^ and *Pla2g12a*^−/−^ mice, IgE/Ag-induced MC degranulation was significantly reduced in *Pla2g12a*^−/−^ mice (Figure 1C,D), indicating that PLA2G12A deficiency impairs MC activation without altering MC abundance. We next assessed systemic anaphylactic responses using an IgE-mediated PSA model. Following systemic Ag challenge, IgE-sensitized *Pla2g12a*^−/−^ mice exhibited attenuated hypothermia and reduced serum histamine levels compared with littermate *Pla2g12a*^+/+^ mice (Figure 1E,F), indicating an attenuated systemic anaphylactic response. By contrast, IgE-independent dye extravasation induced by C48/80, which acts through the G protein-coupled receptor MRGPRB2 [59], was preserved in *Pla2g12a*^−/−^ mice (Figure 1G), suggesting that PLA2G12A preferentially contributes to IgE/Ag-dependent MC activation. Unlike *Pla2g3* deficiency, which impaired MC maturation [12,29,31], dermal MCs in *Pla2g12a*^−/−^ mice exhibited normal expression of c-Kit, FcεRIα, and several other canonical MC marker genes, together with unaltered tissue histamine content and MC protease activities (Figure 1H–K and Figure S2), indicating that *Pla2g12a* deficiency does not influence MC maturation. Collectively, these findings support a role for PLA2G12A in amplifying FcεRI-dependent MC activation and anaphylactic responses in vivo, without affecting MC abundance, maturation, or MRGPRB2-dependent responses.

### 3.2. Fibroblast-Derived PLA2G12A Augments IgE/Ag-Dependent MC Activation In Vivo

**Figure 2:**
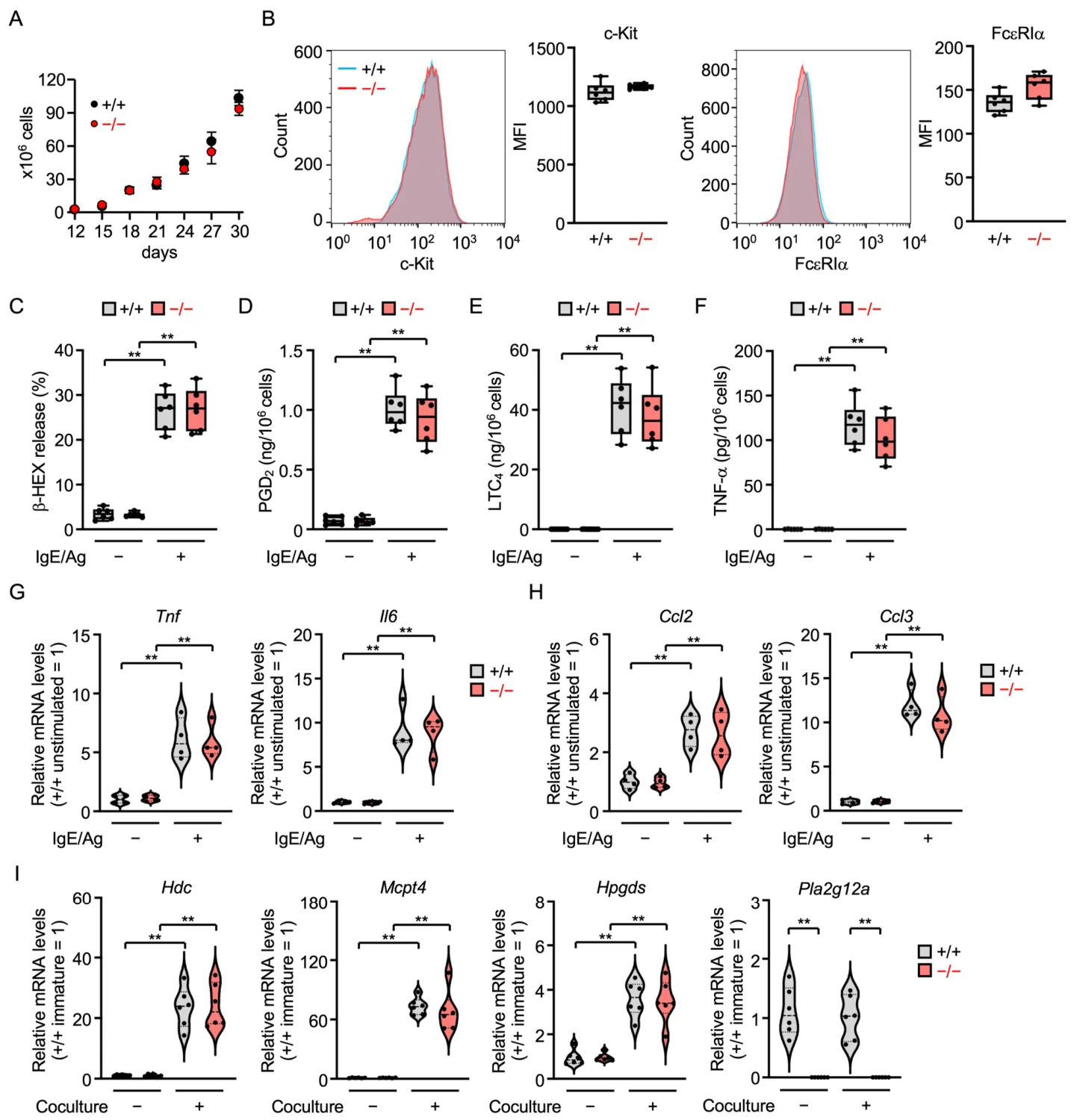
PLA2G12A deficiency does not impair MC activation or fibroblast-dependent maturation in vitro. (**A**) IL-3-dependent proliferation of *Pla2g12a*^+/+^ and *Pla2g12a*^−/−^ BMMCs during in vitro culture. (**B**) Flow cytometric analysis of c-Kit and FcεRIα expression in *Pla2g12a*^+/+^ and *Pla2g12a*^−/−^ BMMCs. (**C**–**F**) IgE/Ag-induced activation of *Pla2g12a*^+/+^ and *Pla2g12a*^−/−^ BMMCs. β-hexosaminidase (β-HEX) release (**C**), PGD_2_ production (**D**), LTC_4_ production (**E**), and TNF-α secretion (**F**) were assessed with or without IgE/Ag stimulation. (**G**,**H**) RT-qPCR analysis of cytokine- and chemokine-associated genes in IgE/Ag-stimulated *Pla2g12a*^+/+^ and *Pla2g12a*^−/−^ BMMCs. Expression levels were normalized to unstimulated *Pla2g12a*^+/+^ BMMCs, which were set to 1. (**I**) RT-qPCR analysis of MC maturation-associated genes in *Pla2g12a*^+/+^ and *Pla2g12a*^−/−^ BMMCs cultured for 4 days with or without Swiss 3T3 fibroblasts in the presence of SCF. Expression levels were normalized to immature *Pla2g12a*^+/+^ BMMCs, which were set to 1. Data are presented as mean ± s.e.m. Representative flow cytometry plots from one of two independent experiments are shown in (**B**). Quantitative data are pooled from two independent experiments. Statistical analysis was performed using a two-tailed Mann–Whitney U test (**B**), Brown–Forsythe and Welch ANOVA followed by Dunnett’s T3 multiple comparisons test (**C**–**I**), or two-way ANOVA followed by Šídák’s multiple comparisons test (**A**). **, *p* < 0.01.

We next examined whether PLA2G12A regulates MC function in a cell-autonomous manner. BMMCs prepared from *Pla2g12a*^−/−^ mice proliferated normally and expressed comparable levels of c-Kit and FcεRIα to those from *Pla2g12a*^+/+^ mice (Figure 2A,B). IgE/Ag-induced degranulation, production of lipid mediators (PGD_2_ and LTC_4_), and secretion of TNF-α were comparable between *Pla2g12a*^+/+^ and *Pla2g12a*^−/−^ BMMCs (Figure 2C–F). Likewise, induction of inflammatory gene expression (e.g., *Tnf*, *Il6*, *Ccl2*, and *Ccl3*) following IgE/Ag stimulation was unaffected by *Pla2g12a* deficiency (Figure 2G,H). Moreover, *Pla2g12a*^−/−^ BMMCs underwent normal maturation following coculture with Swiss3T3 fibroblasts, as assessed by the expression of maturation-inducible genes, including *Hdc*, *Mcpt4*, and *Hpgds* (Figure 2I) [12,29]. These results indicate that MC-intrinsic PLA2G12A is dispensable for both MC activation and maturation.

**Figure 3:**
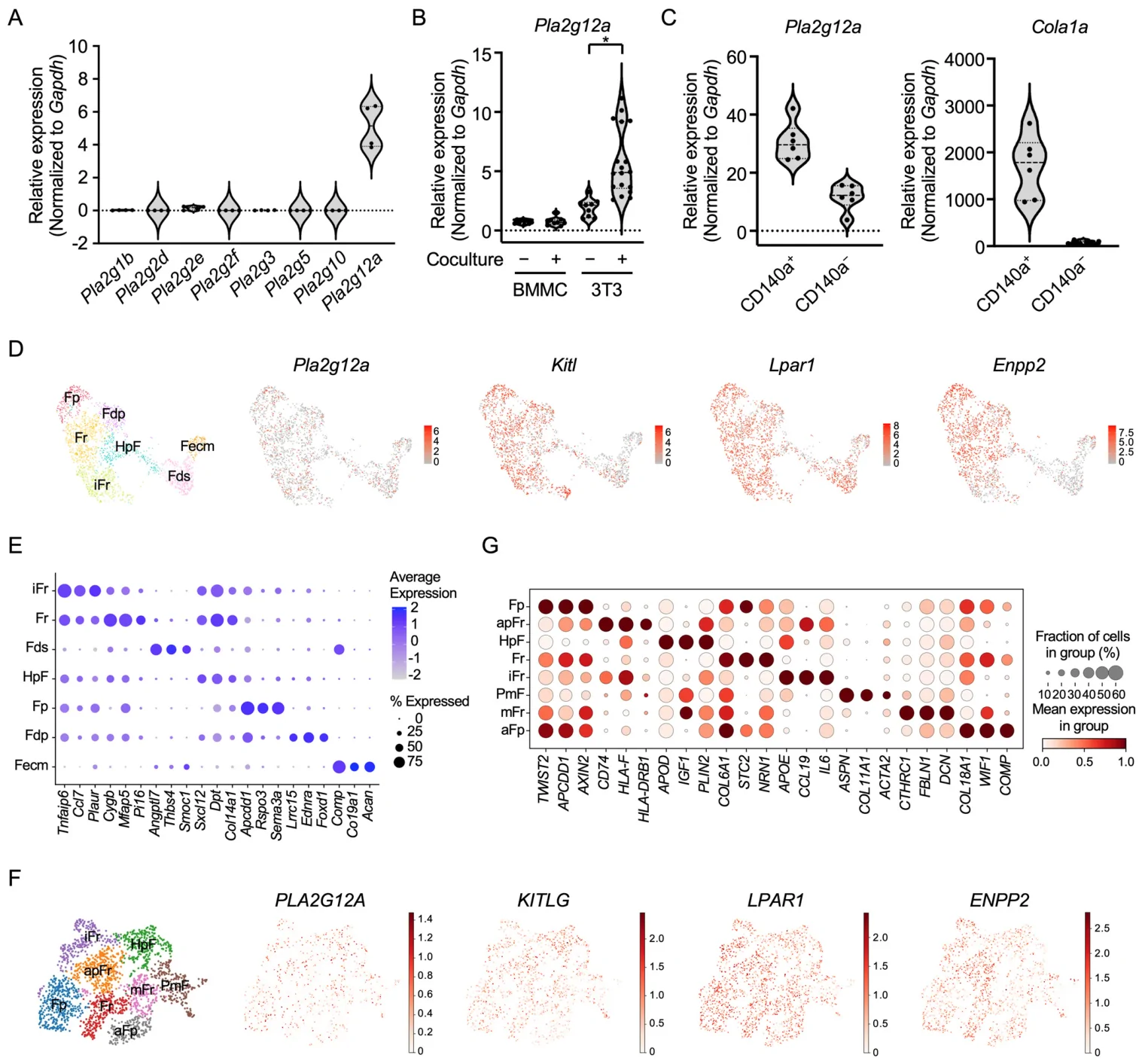
PLA2G12A is broadly expressed in various fibroblast subsets in mouse and human skin. (**A**) Relative mRNA expression of sPLA_2_ isoforms in Swiss 3T3 fibroblasts, as determined by RT-qPCR. Expression levels were normalized to *Gapdh*. (**B**) RT-qPCR analysis of *Pla2g12a* expression in BMMCs and Swiss 3T3 fibroblasts cultured alone or in coculture. Expression levels were normalized to *Gapdh*. (**C**) RT-qPCR analysis of *Pla2g12a* and the fibroblast marker *Col1a1* in CD140a^+^ and CD140a^−^ fractions isolated from mouse ear skin. Expression levels were normalized to *Gapdh*. (**D**) UMAP visualization of annotated fibroblast subsets from our previously reported mouse skin scRNA-seq data (GSE262558), together with feature plots showing expression of *Pla2g12a*, *Kitl*, *Lpar1*, and *Enpp2*. Fibroblast subsets are annotated as follows: Fp, papillary fibroblast; Fr, reticular fibroblast; iFr, activated inflammatory reticular dermal fibroblast; Fdp, follicular dermal papilla/arrector pili-associated fibroblast; Fds, follicular dermal sheath fibroblast; HpF, hypodermal preadipocyte-like fibroblast. (**E**) Dot plot showing expression of fibroblast subset marker genes across the mouse fibroblast subsets shown in (**D**). (**F**) UMAP visualization of fibroblast subsets from a published human skin scRNA-seq data (GSE241132), together with feature plots showing expression of *PLA2G12A*, *KITLG*, *LPAR1*, and *ENPP2*. (**G**) Dot plot showing expression of fibroblast subset marker genes across the human fibroblast subsets shown in (**F**). Data are presented as mean ± s.e.m. Data are pooled from one (**D**,**E**), two (**A**,**C**), or three (**B**) independent experiments. Human scRNA-seq data shown in (**F**,**G**) were generated by reanalyzing a published dataset. Statistical analysis was performed using two-tailed Student’s *t*-test (**B**). *, *p* < 0.05.

These observations prompted us to investigate whether PLA2G12A expressed in non-MCs in tissue microenvironments would be responsible for the regulation of MC responses. *Pla2g12a* was the most abundant sPLA_2_ isoform in Swiss 3T3 fibroblasts (Figure 3A) and was expressed at higher levels than in BMMCs (Figure 3B). Notably, *Pla2g12a* expression in fibroblasts was further increased after coculture with BMMCs (Figure 3B). Consistent with this, *Pla2g12a* expression was enriched in CD140a^+^ dermal fibroblasts compared with CD140a^−^ cells in mouse skin, where enrichment of the fibroblast marker *Col1a1* in the CD140a^+^ fraction confirmed successful fibroblast isolation (Figure 3C). To identify the fibroblast population expressing *Pla2g12a* in vivo, we reanalyzed our previously reported mouse skin scRNA-seq dataset [12]. *Pla2g12a* was broadly expressed in various fibroblast subsets, including a papillary fibroblast subset (Fp) characterized by high expression of *Kitl*, *Lpar1*, and *Enpp2*, consistent with our previous identification of this niche-associated fibroblast population as a regulator of dermal MC maturation and activation (Figure 3D,E) [12]. Furthermore, reanalysis of a published human skin scRNA-seq dataset [51,52] revealed a similar expression pattern, with *PLA2G12A* broadly expressed across multiple fibroblast subsets, including a papillary fibroblast subset characterized by high expression of *KITLG*, *LPAR1*, and *ENPP2* (Figure 3F,G). These findings indicate that PLA2G12A is broadly expressed across fibroblast subsets, including the MC-associated papillary fibroblast population, in both mouse and human skin.

**Figure 4:**
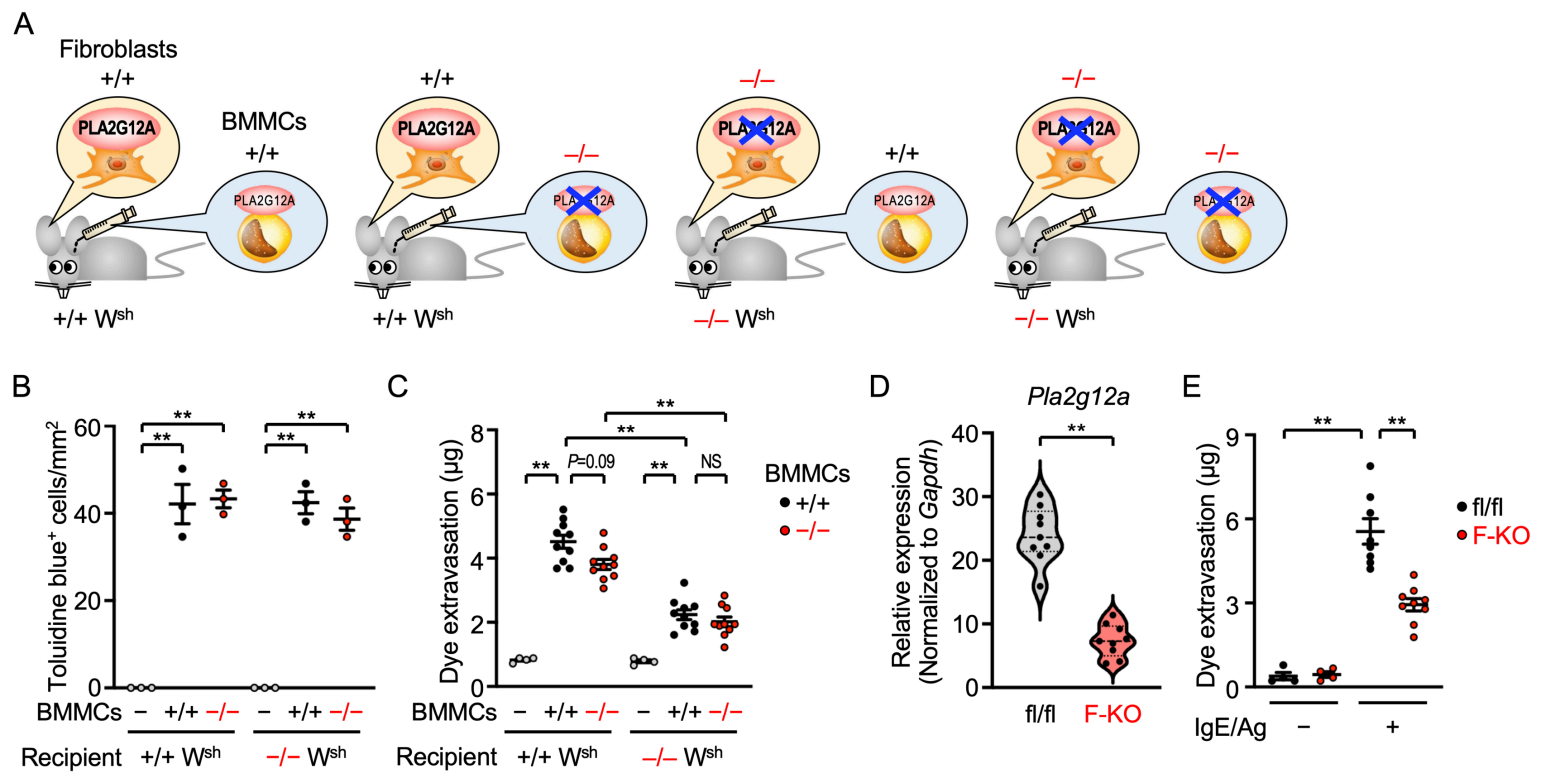
Fibroblast-derived PLA2G12A promotes IgE/Ag-dependent MC activation in vivo. (**A**) Schematic overview of MC reconstitution experiments using *Pla2g12a*^+/+^*Kit^W-sh/W-sh^* (+/+W^sh^) and *Pla2g12a*^−/−^W^sh^ (–/–W^sh^) recipient mice engrafted with *Pla2g12a*^+/+^ or *Pla2g12a*^−/−^ BMMCs. Blue crosses indicate PLA2G12A deficiency. (**B**) Quantification of toluidine blue-positive dermal MCs in ear skin 6 weeks after BMMC engraftment. (**C**) IgE/Ag-induced PCA responses, as assessed by Evans blue extravasation, in MC-reconstituted mice. (**D**) RT-qPCR analysis of *Pla2g12a* expression in ear skin from *Pla2g12a*^fl/fl^ (fl/fl) and fibroblast-targeted *Pla2g12a*-deficient (F-KO) mice generated using *Col1a2*-Cre mice. Expression levels were normalized to *Gapdh*. (**E**) IgE/Ag-induced PCA responses, as assessed by Evans blue extravasation, in fl/fl and F-KO mice. Data are presented as mean ± s.e.m. Data are pooled from one (**B**), two (**D**) or three (**C**,**E**) independent experiments. Statistical analysis was performed using Mann–Whitney U test (**D**), or Brown–Forsythe and Welch ANOVA followed by Dunnett’s T3 multiple comparisons test (**B**,**C**,**E**). **, *p* < 0.01; NS, not significant.

To distinguish the relative contributions of MC-intrinsic and stromal-derived PLA2G12A in vivo, we performed MC reconstitution experiments using *Kit^W-sh/W-sh^* mice [45] crossed onto either *Pla2g12a*^+/+^ or *Pla2g12a*^−/−^ background (Figure 4A). These recipient mice were engrafted with either *Pla2g12a*^+/+^ or *Pla2g12a*^−/−^ BMMCs. Six weeks after engraftment, toluidine blue staining confirmed comparable dermal MC reconstitution across all groups (Figure 4B). In *Pla2g12a*^+/+^*Kit*^W-sh/W-sh^ recipients (+/+W^sh^), transfer of *Pla2g12a*^+/+^ or *Pla2g12a*^−/−^ BMMCs restored PCA responses to comparable levels, indicating that MC-intrinsic PLA2G12A is dispensable for IgE/Ag-dependent MC activation in vivo. By contrast, PCA responses were markedly reduced in *Pla2g12a*^−/−^*Kit^W-sh/W-sh^* recipients (–/–W^sh^) reconstituted with either *Pla2g12a*^+/+^ or *Pla2g12a*^−/−^ BMMCs to a similar level (Figure 4C). These findings indicate that stromal PLA2G12A, rather than MC-intrinsic PLA2G12A, plays a dominant role in promoting IgE/Ag-dependent MC activation in vivo.

To directly test the contribution of fibroblast-derived PLA2G12A, we generated fibroblast-targeted *Pla2g12a*-deficient mice by crossing *Pla2g12a*^fl/fl^ mice with *Col1a2*-Cre mice, in which Cre recombinase is expressed broadly in fibroblasts. Efficient deletion was confirmed by markedly reduced *Pla2g12a* expression in ear skin (Figure 4D). Furthermore, *Col1a2*-Cre-mediated deletion of *Pla2g12a* significantly attenuated PCA responses, phenocopying the defect observed in global *Pla2g12a*^−/−^ mice (Figure 4E). Collectively, these findings demonstrate that fibroblast-derived PLA2G12A is mainly responsible for augmenting IgE/Ag-dependent MC activation in vivo.

### 3.3. Comprehensive Lipidomic Profiling Reveals PLA2G12A-Dependent Changes in Lysophospholipids During IgE/Ag-Induced Anaphylaxis

**Figure 5:**
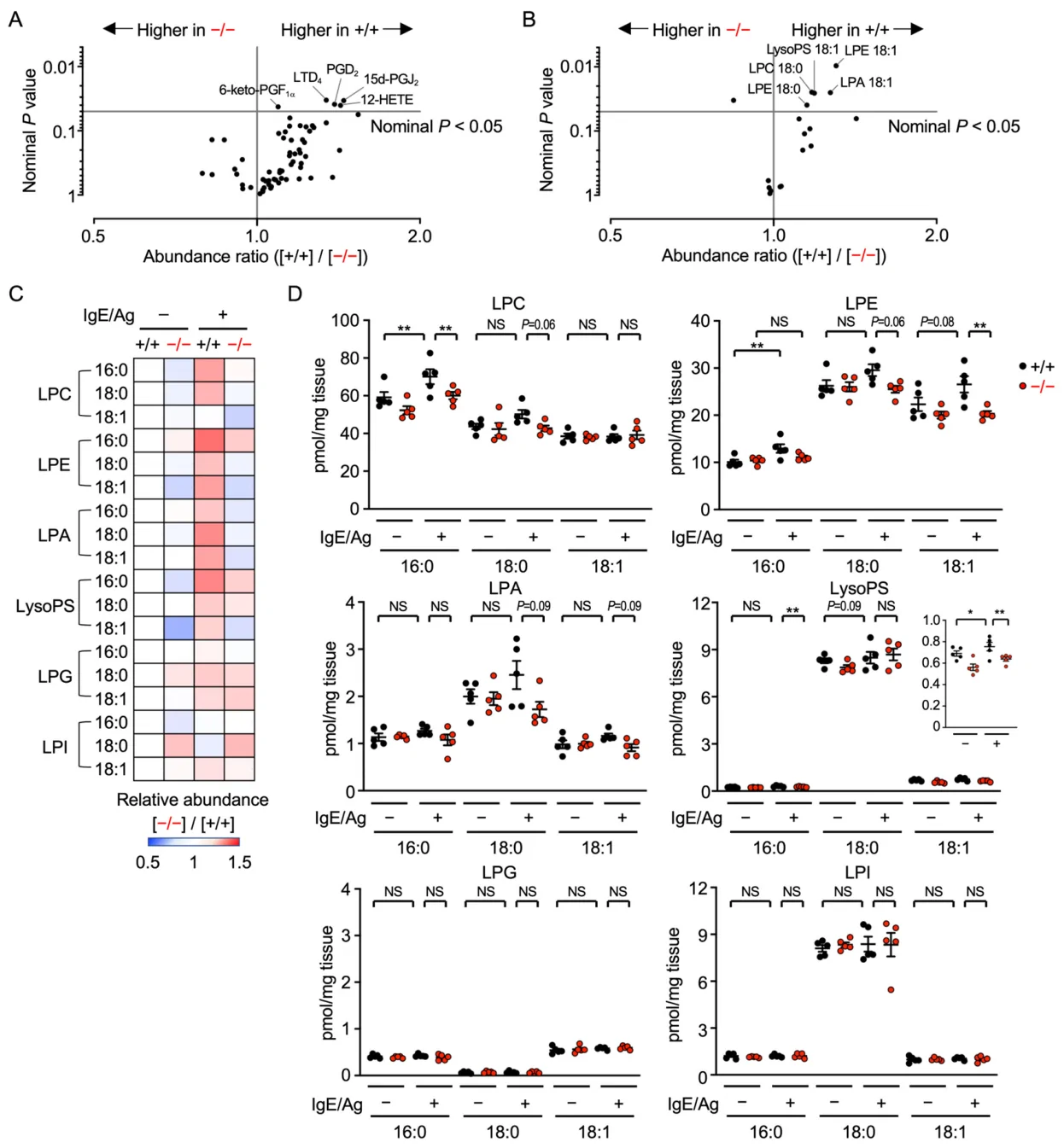
PLA2G12A deficiency reduces lysophospholipid accumulation during IgE/Ag-induced anaphylaxis. (**A**,**B**) Volcano plots showing differential abundance of free fatty acids and oxylipins (**A**) and lysophospholipids (**B**) in ear skin from *Pla2g12a*^+/+^ and *Pla2g12a*^−/−^ mice 2 min after Ag challenge during PCA. Each dot represents an individual lipid species. The *x*-axis indicates the abundance ratio of *Pla2g12a*^+/+^ relative to *Pla2g12a*^−/−^ mice, with values > 1 indicating higher abundance in *Pla2g12a*^+/+^ mice and values < 1 indicating higher abundance in *Pla2g12a*^−/−^ mice. The vertical and horizontal lines indicate a ratio of 1 and a nominal *p* value of 0.05, respectively. (**C**) Heatmap showing the relative abundance of lysophospholipid species in ear skin from *Pla2g12a*^+/+^ and *Pla2g12a*^−/−^ mice immediately before or 2 min after Ag challenge. Colors indicate the relative abundance of individual lysophospholipid species, normalized to the corresponding level in unstimulated *Pla2g12a*^+/+^ mice. Numbers (e.g., 16:0, 18:0, and 18:1) indicate the *sn*-1 acyl chain of each lysophospholipid species. (**D**) Quantification of individual lysophospholipid species in ear skin under the indicated conditions. Insets show enlarged views of LysoPS 18:1. Data are presented as mean ± s.e.m. Data are representative of two independent experiments with similar results. For (**A**,**B**), nominal *p* values were calculated using unpaired two-tailed Student’s *t*-test and adjusted separately for multiple testing in the oxylipin and lysophospholipid datasets using the Benjamini–Hochberg FDR procedure. No lipid species remained significant after FDR correction. For (**D**), statistical analysis was performed using two-way ANOVA to evaluate the effects of genotype, IgE/Ag challenge, and their interaction, followed by Šídák’s multiple-comparisons test. *, *p* < 0.05; **, *p* < 0.01; NS, not significant.

Given the emerging view that sPLA_2_s act on extracellular phospholipids to mobilize bioactive lipid mediators in tissue microenvironments, we attempted to identify such lipid mediators potentially regulated downstream of PLA2G12A during IgE/Ag-induced MC activation. To this end, we performed comprehensive lipidomic analyses of ear skin. Because the effects of *Pla2g12a* deficiency became evident only after Ag challenge, we focused our lipidomic analysis on the immediate phase of the PCA response. Lipids were extracted from ear skin just before or 2 min after Ag challenge, corresponding to the onset of rapid MC degranulation, and subjected to comprehensive analyses of lipid metabolites potentially involved in PLA_2_-dependent phospholipid metabolism, including lysophospholipids, free fatty acids, and their oxygenated metabolites (oxylipins). Volcano plots revealed an overall tendency toward lower levels of various lipid metabolites in *Pla2g12a*^−/−^ mice than in *Pla2g12a*^+/+^ mice following IgE/Ag challenge (Figure 5A,B). Although several lipid species showed nominal differences between genotypes (*p* < 0.05), none remained significant after FDR correction for multiple testing. Among the oxylipins, several AA metabolites (eicosanoids), including the MC-derived lipid mediators PGD_2_ and LTD_4_, showed lower levels in Ag-challenged *Pla2g12a*^−/−^ skin compared with *Pla2g12a*^+/+^ skin (Figure 5A). These changes, even though relatively modest, are consistent with the attenuated MC activation observed in *Pla2g12a*^−/−^ mice and may contribute partly to the reduced vascular permeability during PCA. Compared to these oxylipins, various lysophospholipid species were more profoundly affected by PLA2G12A deficiency (Figure 5B). Heatmap analysis showed that Ag challenge induced coordinated increases in multiple lysophospholipid species in *Pla2g12a*^+/+^ mice, whereas these responses were broadly attenuated in *Pla2g12a*^−/−^ mice (Figure 5C). Quantitative analyses further showed that, despite their high background levels in tissues, several LPC, LPE, and LPA species were elevated in *Pla2g12a*^+/+^ skin following Ag challenge, whereas these increases were attenuated in *Pla2g12a*^−/−^ skin (Figure 5D). Among the other lysophospholipids, LysoPS 18:1 also tended to be lower in *Pla2g12a*^−/−^ skin after and even before Ag challenge, while lysophosphatidylglycerol (LPG) and lysophosphatidylinositol (LPI) species were largely unaffected by *Pla2g12a* deficiency (Figure 5C,D). These results suggest that one or more of these lysophospholipids may act downstream of PLA2G12A during IgE/Ag-induced anaphylaxis to amplify MC activation.

### 3.4. PLA2G12A Generates Extracellular Lysophospholipids from Phospholipids in EV-Enriched Fractions and Restores Impaired PCA Responses in Pla2g12a-Deficient Mice

**Figure 6:**
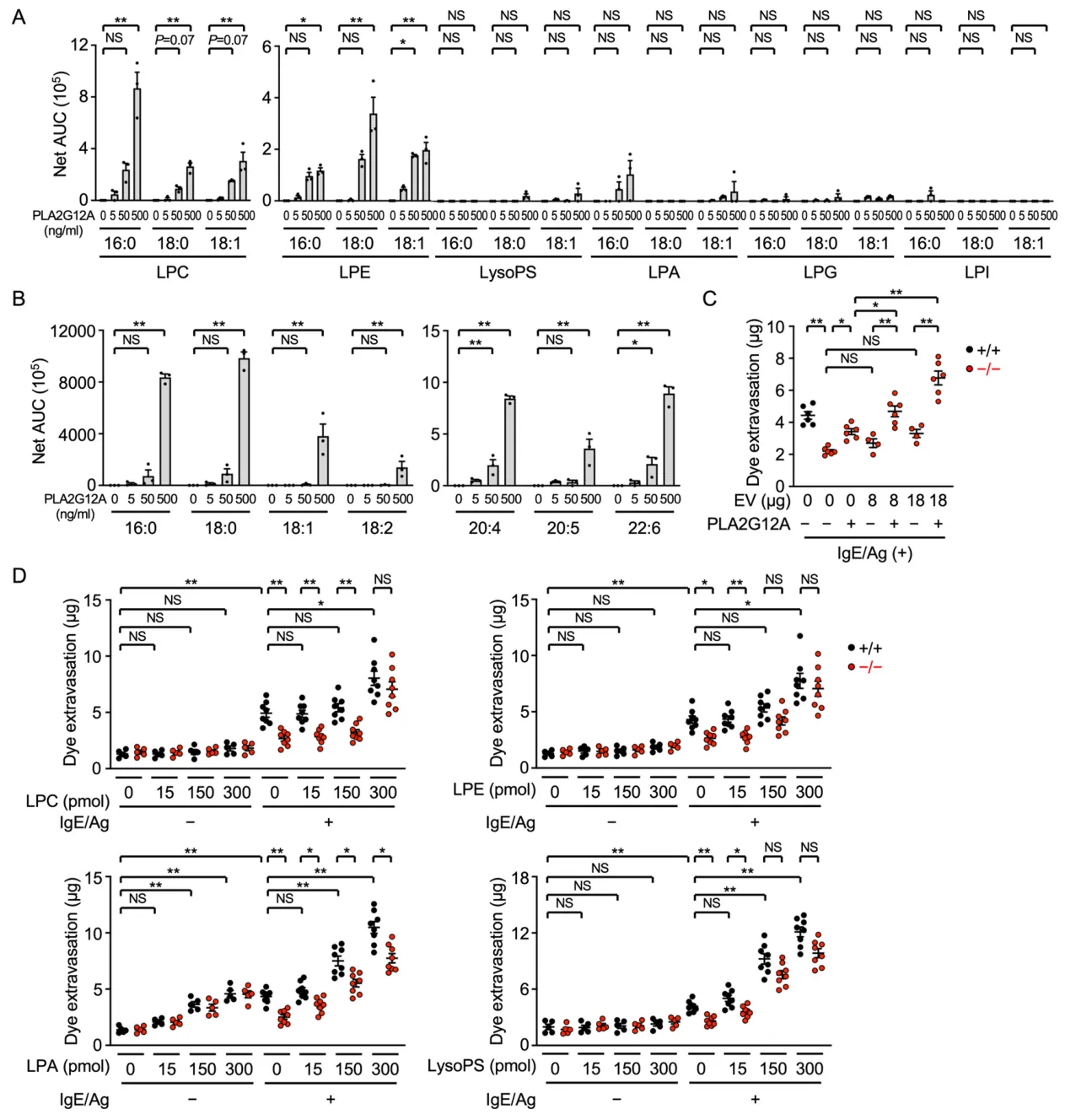
PLA2G12A generates multiple lysophospholipids from EV-enriched fractions and restores impaired PCA responses in *Pla2g12a*-deficient mice. (**A**,**B**) Lipidomic analysis of lysophospholipids (**A**) and free fatty acids (**B**) generated in EV-enriched fractions prepared from the supernatants of SCF-dependent cocultures of BMMCs and Swiss 3T3 fibroblasts following incubation with recombinant PLA2G12A at the indicated concentrations. In (**A**), 16:0, 18:0, and 18:1 indicate the *sn*-1 acyl chain of individual lysophospholipid species, whereas in (**B**), 16:0, 18:0, 18:1, 18:2, 20:4, 20:5, and 22:6 indicate the corresponding free fatty acid species. Net LC–MS peak areas (AUC) are shown. (**C**) IgE/Ag-induced PCA responses in *Pla2g12a*^+/+^ and *Pla2g12a*^−/−^ mice following intradermal administration of PLA2G12A alone or EV-enriched fractions, with (+) or without (−) PLA2G12A pretreatment just before Ag challenge. Vascular permeability was quantified by Evans blue dye extravasation. (**D**) IgE/Ag-induced PCA responses in *Pla2g12a*^+/+^ and *Pla2g12a*^−/−^ mice following intradermal administration of the indicated doses of LPC 18:1, LPE 18:1, LPA 18:1, or LysoPS 18:1 immediately before Ag challenge. Evans blue dye extravasation was quantified as an index of vascular permeability. Data are presented as mean ± s.e.m. Data are representative of two independent experiments with similar results (**A**,**B**) or pooled from two independent experiments (**C**,**D**). Statistical analysis was performed using ordinary one-way ANOVA followed by Šídák’s multiple comparisons test (**A**,**B**) or Brown–Forsythe and Welch ANOVA followed by Dunnett’s T3 multiple comparisons test (**C**,**D**). *, *p* < 0.05; **, *p* < 0.01; NS, not significant.

We have recently shown that PLA2G12A hydrolyzes phospholipids in EVs released from activated T cells, resulting in the extracellular generation of lysophospholipids that promote Th17 differentiation [14]. To determine whether a similar mechanism operates in MC microenvironments, EV-enriched fractions were prepared from SCF-dependent cocultures of BMMCs and Swiss 3T3 fibroblasts using an EV isolation kit and incubated with recombinant PLA2G12A. Lipidomic analysis revealed dose-dependent generation of multiple lysophospholipid species following PLA2G12A treatment, with LPC and LPE representing the major products, whereas other lysophospholipid classes were generated at lower absolute levels despite showing dose-dependent increases (Figure 6A). Concomitantly, PLA2G12A treatment increased nonselective release of multiple free fatty acids (Figure 6B). These results confirm that PLA2G12A hydrolyzes phospholipids in EV-enriched fractions, particularly phosphatidylcholine (PC) and phosphatidylethanolamine (PE), generating multiple lysophospholipid species, including LPC and LPE.

We next examined whether PLA2G12A-modified EV-enriched fractions could restore the impaired PCA response observed in *Pla2g12a*^−/−^ mice. While intradermal administration of PLA2G12A alone restored IgE/Ag-induced vascular leakage only partially in *Pla2g12a*^−/−^ mice, PLA2G12A-treated EV-enriched fractions restored it much more efficiently in a dose-dependent manner to levels comparable to those in *Pla2g12a*^+/+^ mice (Figure 6C). PLA2G12A-untreated EV-enriched fractions did not significantly restore the response at the corresponding amounts. Of note, although the same amount (9 ng) of PLA2G12A was carried over in different doses of the PLA2G12A-treated EV groups, they showed a greater and synergistic rescue, particularly at a higher dose. These observations suggest that lysophospholipids generated through PLA2G12A-mediated hydrolysis of phospholipids in EV-enriched fractions contribute to the restoration of the impaired PCA response in *Pla2g12a*^−/−^ mice. To determine whether the major lysophospholipid species identified by lipidomic analyses indeed contributed to this activity, the lysophospholipid species generated following PLA2G12A treatment of EV-enriched fractions were administered intradermally immediately before Ag challenge. We injected 15–300 pmol of lysophospholipids, with the doses selected based on their endogenous amounts detected in the skin. When these lysophospholipids were administered to *Pla2g12a*^+/+^ mice, LysoPS augmented the PCA response most effectively, showing its activity at concentrations of as low as 150 pmol, followed by LPE and LPC, which were active only at 300 pmol. The attenuated PCA response in *Pla2g12a*^−/−^ mice was restored to levels comparable to *Pla2g12a*^+/+^ mice by LysoPS and LPE (at 150 pmol) and by LPC (at 300 pmol). None of these lysophospholipids increased dye extravasation when administered alone. Thus, all of these lysophospholipids augmented the IgE/Ag-induced PCA response, with a potency order of LysoPS > LPE > LPC (Figure 6D). LPA displayed a distinct activity profile, increasing vascular leakage irrespective of *Pla2g12a* genotype and even in the absence of Ag challenge, resulting in only partial normalization of the attenuated PCA response in *Pla2g12a*^−/−^ mice (Figure 6D). These results suggest that individual lysophospholipid species have the capacity to augment the PCA response with distinct potencies, collectively contributing to the amplification of IgE/Ag-induced anaphylactic responses downstream of PLA2G12A-mediated hydrolysis of phospholipids in EV-enriched fractions. Considering the mass abundance and selectivity of individual lysophospholipids generated by PLA2G12A in tissues (Figure 5D) and EV-enriched fractions (Figure 6A), LPE and/or LPC are likely to make major contributions to this response. Given that the amounts of lysophospholipids generated by PLA2G12A pretreatment were estimated to be only a few pmol per 10 μg of EV-enriched fractions, the simultaneous generation of multiple lysophospholipid species in EV membranes appears to be far more effective than lysophospholipids alone in augmenting MC activation. These results collectively support PLA2G12A-dependent extracellular phospholipid remodeling as a mechanism that amplifies IgE/Ag-induced MC activation.

## 4. Discussion

In the present study, we identify PLA2G12A as a previously unrecognized stromal regulator of IgE/Ag-dependent MC activation. Whereas previous studies have shown that several sPLA_2_ isoforms can enhance MC degranulation when overexpressed or added exogenously at relatively high concentrations in vitro [12,29,33,34,60], the in vivo relevance of these observations had remained elusive. Our present findings have demonstrated that endogenous PLA2G12A secreted by stromal fibroblasts physiologically regulates immediate allergic responses in vivo. Mechanistically, PLA2G12A hydrolyzes extracellular phospholipids, likely EV membrane phospholipids, thereby generating bioactive lysophospholipids that enhance IgE/Ag-dependent MC activation. These findings provide an additional line of evidence that EV membrane phospholipids serve as physiologically relevant extracellular substrates for sPLA_2_s (in this case PLA2G12A), although other extracellular phospholipid structures possibly present in the EV-enriched fraction, such as lipoproteins, dying cells, and membrane debris, may also serve as a hydrolytic platform for sPLA_2_s in vivo [43,61,62,63,64]. Collectively, our study establishes stromal PLA2G12A-dependent extracellular phospholipid remodeling as a distinct lipid-driven mechanism through which tissue stromal niches fine-tune MC responsiveness during immediate allergic reactions and expands our understanding of the general role of sPLA_2_-dependent EV modification in various pathophysiological events [10,11,12,13,14,15].

One of the most notable findings of this study is that PLA2G12A regulated FcεRI-dependent, but not MRGPRB2-dependent, MC activation. This property is reminiscent of the responsiveness of rodent CTMCs to lysophospholipids, particularly LysoPS, which augments IgE/Ag-induced MC activation rather than directly inducing it [65,66]. This selectivity provides an important clue to the underlying mechanism of lysophospholipid action on MCs. Unlike MRGPRB2, which primarily activates Gα_q_/PLCβ-dependent Ca^2+^ signaling [67], FcεRI signaling critically depends on the assembly of LAT-centered signaling complexes within cholesterol-rich membrane microdomains before PLCγ activation and downstream degranulation [68,69]. We therefore speculate that extracellular lysophospholipids generated downstream of PLA2G12A do not function as direct MC agonists by acting on their cognate GPCRs, but instead lower the activation threshold of FcεRI signaling. Owing to their cone-shaped amphipathic structures, lysophospholipids can modify membrane curvature, lipid packing, and the lateral organization of the plasma membrane [70,71]. These biophysical changes could influence the dynamics of these membrane microdomains, thereby facilitating the assembly or stabilization of FcεRI-associated signaling complexes and lowering the threshold for Ag-induced MC activation, while exerting relatively little influence on GPCR-mediated MRGPRB2 signaling. In addition to these membrane biophysical effects, lysophospholipids may also potentiate FcεRI signaling by enhancing store-operated Ca^2+^ entry (SOCE). Sustained Ca^2+^ influx through STIM1/Orai1-dependent SOCE is indispensable for FcεRI-dependent MC degranulation, whereas C48/80-induced (i.e., MRGPRB2-dependent) MC activation depends solely on Ca^2+^ release from the intracellular store and does not require extracellular Ca^2+^ influx [72,73,74]. Consistent with this possibility, current studies have demonstrated that lysophospholipids generated by PLA2G6 (iPLA_2_β), a member of the patatin-like phospholipase domain-containing (PNPLA) family, facilitate Orai1-dependent SOCE in muscles and neurons [75,76,77], suggesting that lysophospholipids themselves can promote sustained Ca^2+^ influx. Although it remains unknown whether SOCE in MCs is similarly regulated by lysophospholipids supplied extracellularly by PLA2G12A, this mechanism provides an attractive hypothesis linking sPLA_2_-mediated extracellular production of lysophospholipids to FcεRI signaling.

Reportedly, the enhancement of MC activation by lysophospholipids is dependent on FcεRI, but not MRGPRB2, and occurs specifically in CTMCs rather than BMMCs, recapitulating the action of PLA2G12A observed in this study. Although LysoPS has long been recognized as the most potent lysophospholipid to augment CTMC activation [25], several lysophospholipid species other than LysoPS, including LPC and LPA, can also potentiate MC activation [12,26,78], and our present reevaluation, employing multiple lysophospholipids, has delineated that various lysophospholipids have the capacity to augment the PCA response downstream of PLA2G12A, with the potency order of LysoPS > LPE > LPC. Exceptionally, the action of LPA is unique, showing low PLA2G12A dependence. Taken together, our results support a model in which multiple lysophospholipid species generated by stromal PLA2G12A collectively amplify FcεRI signaling. Notably, the robust rescue achieved with PLA2G12A-treated EV-enriched fractions, despite the relatively small amounts of lysophospholipids estimated to be generated, suggests that PLA2G12A-dependent phospholipid remodeling within EVs or related extracellular membrane components can enhance MC activation more efficiently than freely administered lysophospholipids. Consistent with our previous findings [12,14], lysophospholipids newly generated by sPLA_2_ may remain associated with extracellular lipid-containing structures, including EV membranes, thereby facilitating and eventually enabling their coordinated presentation to target cells, MCs in this case, in the local stromal niche while limiting their diffusion into the extracellular milieu. EV membranes may therefore serve not only as physiologically relevant extracellular substrates for PLA2G12A but also as platforms that facilitate the presentation of newly generated lysophospholipids to target MCs.

**Figure 7:**
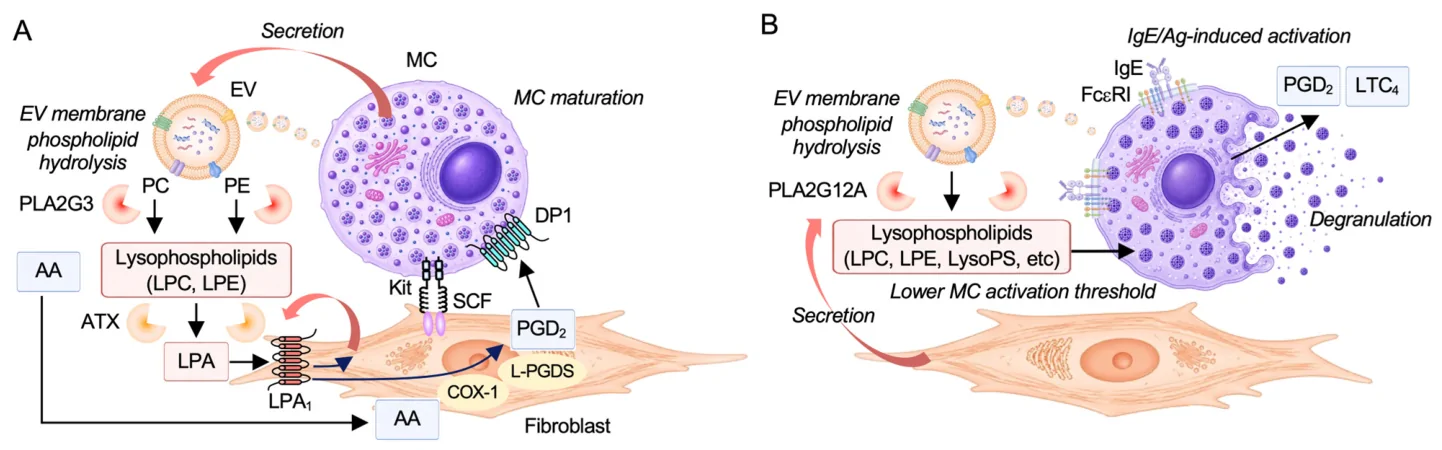
Distinct roles of extracellular phospholipid metabolism in MC biology mediated by PLA2G3 and PLA2G12A. (**A**) MC-derived PLA2G3 hydrolyzes phospholipids in EVs constitutively supplied by maturing MCs (and possibly fibroblasts), generating lysophospholipid intermediates, including LPC and LPE. Through sequential metabolism involving autotaxin (ATX, also known as ENPP2), this lysophospholipid pathway culminates in LPA generation, which activates LPA_1_ receptor on fibroblasts to coordinate multiple maturation-associated signals, including PGD_2_–DP1 signaling, thereby promoting MC maturation over an extended time scale. (**B**) Fibroblast-derived PLA2G12A hydrolyzes phospholipids in EVs supplied by activated MCs to rapidly generate bioactive lysophospholipids, including LPE, LPC, and LysoPS. These lysophospholipids act within minutes to lower the activation threshold of mature MCs, thereby potentiating IgE/FcεRI-dependent activation and degranulation.

The present findings also highlight the functional diversity of sPLA_2_ family members in regulating MC biology. Our previous studies demonstrated that MC-derived PLA2G3 hydrolyzes phospholipids in EVs constitutively supplied from maturing MCs, initiating extracellular phospholipid metabolism that leads to fibroblast-derived PGD_2_ production and supports MC maturation [12,29]. In contrast, fibroblast-derived PLA2G12A regulates the activation threshold of mature MCs through the local generation of lysophospholipids. Since PLA2G12A deficiency cancels the increase in lysophospholipids following IgE/Ag challenge, it is conceivable that PLA2G12A secreted from stromal fibroblasts acts on phospholipid substrates, likely those in EV membranes released from activated MCs in the local niche. Indeed, activated MCs release EVs abundantly, which contribute to the regulation of MCs themselves and neighboring cells [6,7]. Although both the PLA2G3- and PLA2G12A-dependent pathways depend on extracellular lysophospholipid generation and intercellular communication between MCs and fibroblasts, they are distinguished by the cellular source and temporal context of their secretion, the direction of intercellular signaling, and the stage of MC biology (maturation and activation) that they regulate. Collectively, these observations suggest that the biological functions of sPLA_2_s are determined not only by their intrinsic substrate specificity, but also by the spatiotemporal context of their secretion and the lipid composition of their target membranes. This concept supports a broader conceptual framework in which extracellular phospholipid metabolism orchestrates distinct stages and mechanisms of tissue MC biology (Figure 7 and Table 1).

Several limitations and important questions remain to be addressed. Although dermal MC abundance and FcεRI expression were comparable between *Pla2g12a*^+/+^ and *Pla2g12a*^−/−^ mice, these parameters were not systematically assessed in other tissues relevant to systemic anaphylaxis. Thus, we do not fully rule out the possibility that the differences in MC abundance or FcεRI expression in these extra-skin tissues might contribute to the attenuated PSA response in *Pla2g12a*^−/−^ mice. Although our whole-tissue lipidomic analyses revealed PLA2G12A-dependent changes in lysophospholipid levels, these measurements cannot resolve the concentrations or spatial distribution of extracellular *versus* intracellular lysophospholipids in local MC–fibroblast niches. The mechanisms through which individual lysophospholipids regulate MC activation remain incompletely understood. It remains to be determined whether lysophospholipids enhance FcεRI signaling by modulating plasma-membrane microdomains, STIM1/Orai1-dependent SOCE, or additional receptor-dependent or receptor-independent pathways. LPA, a distinct lysophospholipid mediator, may act through distinct MC-dependent and MC-independent mechanisms, including its effects on vascular endothelial functions via six LPA receptors. The identity and physiological relevance of the extracellular phospholipid substrates of PLA2G12A remain to be firmly established. Because the EV-enriched fractions used here were obtained by polymer-based precipitation, our data do not necessarily indicate that EV membrane phospholipids indeed serve as specific substrates for PLA2G12A. Whether EVs released from different MC subsets or in response to distinct activation stimuli are similarly metabolized, and whether PLA2G12A-mediated hydrolysis alters EV cargo delivery or EV biological activities or behaviors, remain unknown. Because recombinant PLA2G12A was not removed or inactivated before administration of PLA2G12A-treated EV-enriched fractions, the contribution of lysophospholipids generated during the ex vivo incubation cannot be distinguished from that of continued PLA2G12A-dependent phospholipid hydrolysis after in vivo administration. Therefore, the rescue observed with these fractions cannot be attributed specifically to pre-generated lysophospholipids. Although PLA2G12A-treated EV-enriched fractions enhanced the PCA response in a dose-dependent manner, whether they directly enhanced MC degranulation or involved some additional mechanisms remains to be determined. Although we specifically focused on anaphylaxis in this study, the functional relevance of PLA2G12A-mediated MC regulation in broader pathophysiological events, including host defense against *Staphylococcus aureus* or parasites, venom detoxification, type 2 immune responses, and other MC-associated tissue homeostasis and disorders, remains to be established. Finally, whether PLA2G12A-dependent extracellular phospholipid hydrolysis similarly regulates MC activation and allergic diseases in humans warrants further investigation. Addressing these questions should provide important insights into the physiological and pathological significance of stromal regulation of MC activation in human tissues and provide opportunities for the development of PLA2G12A-targeted inhibitors or antibodies to treat MC-associated diseases.

## 5. Conclusions

In summary, this work provides a conceptual framework for understanding how stromal regulation of extracellular phospholipid remodeling fine-tunes tissue MC responsiveness. Our findings further highlight a role for fibroblast-derived PLA2G12A in amplifying IgE/Ag-dependent MC activation and immediate allergic responses through extracellular phospholipid remodeling.

## Figures and Tables

**Table 1:** Comparison of the PLA2G3- and PLA2G12A-mediated pathways regulating MC biology through extracellular phospholipid metabolism. The table summarizes the distinct characteristics of the PLA2G3- and PLA2G12A-mediated pathways, including the cellular source of sPLA_2_, physiological timing, extracellular substrates, lipid metabolites, target cells, signaling mechanisms, and biological functions. These pathways illustrate how extracellular phospholipid metabolism, together with the intrinsic substrate specificity, the spatiotemporal context of distinct sPLA_2_s, and the availability and lipid composition of target EVs, regulates different stages of tissue MC biology.

Feature	PLA2G3 Pathway	PLA2G12A Pathway
sPLA_2_ source	MC	Fibroblast
Physiological timing	MC maturation	Immediate allergic reaction
Primary extracellular substrate	EV membrane phospholipids	EV membrane phospholipids
Major bioactive lipid mediator(s)	Lysophospholipid intermediates → LPA (via ATX)	Bioactive lysophospholipids (LPE, LPC, LysoPS)
Initial target cell(s)	Fibroblast	Mature MC
Principal signaling pathway	LPA–LPA_1_-dependent stromal signaling	Direct lysophospholipid-mediated lowering of the FcεRI activation threshold
Primary biological outcome	MC maturation	MC activation
Conceptual role	Niche-mediated MC maturation	Niche-mediated tuning of MC activation

## Data Availability

Publicly available scRNA-seq datasets analyzed in this study are cited in the Materials and Methods. All other data supporting the findings of this study are available from the corresponding authors upon reasonable request.

## Supplementary Materials

The following supporting information can be downloaded at: https://www.mdpi.com/article/10.3390/cells15201848/s1, Figure S1. Additional characterization of IgE/Ag-induced PCA in *Pla2g12a*-deficient mice. Figure S2. Flow cytometric gating strategy for identification of dermal c-Kit^+^FcεRIα^+^ mast cells from mouse ear skin. Table S1. TaqMan Gene Expression Assays used for RT-qPCR.

## Abbreviations

The following abbreviations are used in this manuscript:

AA	Arachidonic acid
Ag	Antigen
ATX	Autotaxin
BMMC	Bone marrow-derived mast cell
CPA	Carboxypeptidase A
cPLA_2_α	Cytosolic phospholipase A_2_α
CTMC	Connective tissue-type mast cell
FDR	False discovery rate
EV	Extracellular vesicle
FcεRI	High-affinity IgE receptor
Fdp	Follicular dermal papilla/arrector pili-associated fibroblast
Fds	Follicular dermal sheath fibroblast
Fp	Papillary fibroblast
HpF	Hypodermal preadipocyte-like fibroblast
iFr	Activated inflammatory reticular dermal fibroblast
LC-MS/MS	Liquid chromatography–tandem mass spectrometry
LPA	Lysophosphatidic acid
LPC	Lysophosphatidylcholine
LPE	Lysophosphatidylethanolamine
LPG	Lysophosphatidylglycerol
LPI	Lysophosphatidylinositol
LT	Leukotriene
LysoPS	Lysophosphatidylserine
MC	Mast cell
MRGPRB2	Mas-related G protein-coupled receptor B2
PAFAH2	Platelet-activating factor acetylhydrolase II
PC	Phosphatidylcholine
PCA	Passive cutaneous anaphylaxis
PE	Phosphatidylethanolamine
PG	Prostaglandin
PSA	Passive systemic anaphylaxis
PUFA	Polyunsaturated fatty acid
RT-qPCR	Reverse transcription-quantitative polymerase chain reaction
SCF	Stem cell factor
scRNA-seq	Single-cell RNA sequencing
SOCE	Store-operated Ca^2+^ entry
sPLA_2_	Secreted phospholipase A_2_
