# Supplementary material for: Fibroblast-Derived PLA2G12A Amplifies Mast Cell Activation Through Extracellular Phospholipid Remodeling
Source: Cells. 2026 Oct 12;15(20):1848. doi: 10.3390/cells15201848 (PMC13702701; doi:10.3390/cells15201848)
Supplement: Supplementary file 1 [file cells-15-01848-s001.zip › cells-4535495-supplementary.pdf]

Supplementary Information

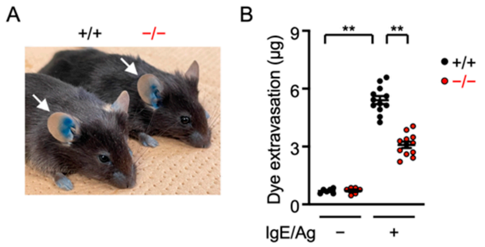

Figure S1. Additional characterization of IgE/Ag-induced PCA in *Pla2g12a*-deficient mice. (A) Representative gross image of the ears 30 min after Ag challenge in male *Pla2g12a*$^{+/+}$ and *Pla2g12a*$^{-/-}$ mice. Arrows indicate Evans blue extravasation. (B) Evans blue extravasation in female *Pla2g12a*$^{+/+}$ and *Pla2g12a*$^{-/-}$ mice with or without IgE/Ag-induced PCA. Data are presented as mean ± s.e.m. A representative image from three independent experiments is shown in (A). Data are pooled from two independent experiments. Statistical analysis was performed using Brown–Forsythe and Welch ANOVA followed by Dunnett's T3 multiple comparisons test. **, $p < 0.01$.

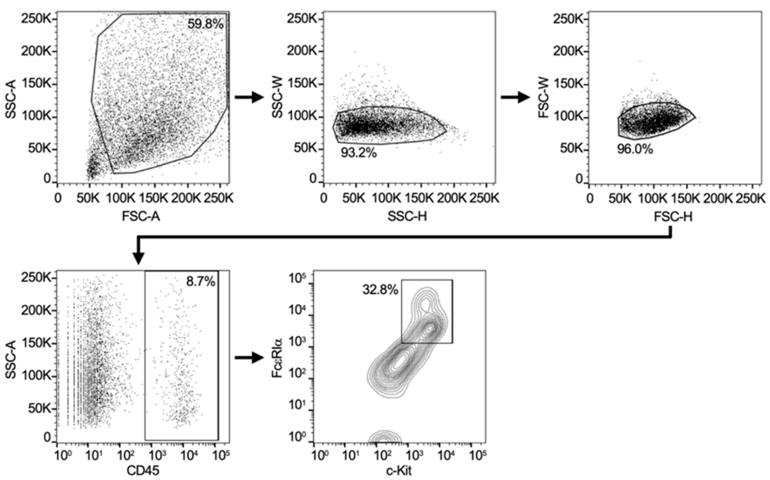

Figure S2. Flow cytometric gating strategy for identification of dermal c-Kit$^+$FcεRIα^+ mast cells from mouse ear skin. Ear skin cells were sequentially gated based on FSC/SSC characteristics, singlets, CD45 positivity, and c-Kit and FcεRIα expression. Representative flow cytometry plots from one experiment are shown.

Gene	Assay ID
Fcer1a	Mm00438867_m1
Kit	Mm00445212_m1
Hdc	Mm00456104_m1
Mcpt4	Mm00487636_g1
Tpsb2	Mm00487645_m1
Cpa3	Mm00483940_m1
Ccl2	Mm00441242_m1
Ccl3	Mm00441258_m1
Il6	Mm00446190_m1
Tnf	Mm00443258_m1
Pla2g1b	Mm00478249_m1
Pla2g2d	Mm00448160_m1
Pla2g2e	Mm00478870_m1
Pla2g2f	Mm00478872_m1
Pla2g3	Mm01191142_m1
Pla2g5	Mm00448162_m1
Pla2g10	Mm00449532_m1
Pla2g12a	Mm01316982_m1
Gapdh	4352339E

Table S1. TaqMan Gene Expression Assays used for RT-qPCR.
